# A Rare Case of Systemic Amyloidosis Involving the Thyroid in a Young Patient

**DOI:** 10.3390/jcm14196741

**Published:** 2025-09-24

**Authors:** Oliwia Julia Kasprzak, Kamila Stępińska, Kaja Kiedrowska, Tomasz Błaszkowski, Aleksandra Kudrymska, Sylwia Sikora, Maciej Miernik, Maciej Romanowski

**Affiliations:** 1 Surgical Scientific Society at the Department of General and Oncological Surgery, Pomeranian Medical University in Szczecin, 71-252 Szczecin, Poland; kamilastepinska2708@gmail.com (K.S.); kajakiedrowska@gmail.com (K.K.); tomasz.blaszkowski@pum.edu.pl (T.B.); dr.maciej.romanowski@gmail.com (M.R.); 2 Department of General and Oncological Surgery, Pomeranian Medical University in Szczecin, 71-252 Szczecin, Poland; maciej.miernik@pum.edu.pl; 3 Department of Pathomorphology, Pomeranian Medical University in Szczecin, 71-252 Szczecin, Poland; akudrymska@gmail.com (A.K.); ssikora95@wp.pl (S.S.)

**Keywords:** amyloidosis, thyroid amyloidosis, thyroid imaging, thyroid surgery, goitre

## Abstract

Thyroid amyloidosis is a rare condition associated with thyroid pathologies such as medullary carcinoma, papillary carcinoma, amyloid goitre, and benign lesions, with a clinically palpable goitre being exceptionally uncommon. As a result, many cases of benign thyroid enlargement caused by amyloid deposits remain undiagnosed. A 28-year-old male patient noticed progressive neck circumference enlargement, voice alteration, decreased appetite, weight loss, dysphagia, fever, and night sweats. Fine-needle aspiration biopsy of the thyroid gland did not reveal the cause of the goitre. A total thyroidectomy was performed. Histopathological examination confirmed advanced thyroid amyloidosis.

## 1. Introduction

The term “amyloid” was first coined in the 19th century by Matthias Schleiden (1804–1881), a German botanist [1], and was subsequently introduced into the medical literature in 1853 by Rudolf Virchow (1821–1902), a German pathologist [1,2]. Amyloidosis is a rare, multifactorial, and heterogeneous group of disorders characterised by the extracellular deposition of insoluble fibrillar proteins—known as amyloid—in various tissues and organs, ultimately leading to their dysfunction [1,3,4,5].

AA amyloidosis, historically referred to as secondary amyloidosis, arises as a complication of chronic inflammatory or autoimmune conditions, and less commonly, malignancies [6]. Epidemiological data from the periods 1956–2005 and 2000–2009 reveal a decline in amyloidosis cases associated with infections and inflammatory diseases, alongside an increase in those linked to autoimmune and neoplastic conditions [7].

The kidneys are the most frequently affected organs [6], although amyloid deposits may also involve the heart, liver, and endocrine system—particularly the thyroid gland and testes—resulting in organ enlargement and functional impairment [3]. The presence of amyloid in the thyroid is commonly associated with medullary thyroid carcinoma [3,5], and secondary amyloidosis should therefore be considered in the differential diagnosis of this malignancy [5]. In some cases, amyloid deposition is also observed in other thyroid pathologies, including papillary thyroid carcinoma, amyloid goitre, and benign lesions [3].

Clinical presentation depends on the anatomical distribution of amyloid deposits [6]. Amyloid goitre may remain indolent over several years or enlarge rapidly, leading to acute symptoms. The most common clinical features include compressive symptoms and thyroid dysfunction (either hypothyroidism or hyperthyroidism), although patients may also report persistent hoarseness, orthopnoea, and cosmetic concerns [8].

Diagnosis requires fine-needle aspiration biopsy (FNAB) [6,9], with histopathological confirmation of amyloid deposits [6]. Congo red staining remains the gold standard, confirming amyloid by its characteristic apple-green birefringence under polarised light [5,6]. Microscopically, thyroid parenchyma is partially replaced by amyloid and adipose tissue [9]. Macroscopically, the thyroid may appear normal or significantly enlarged—up to twice its normal size—and demonstrates a firm, whitish to pale pink cut surface due to amorphous amyloid accumulation [5]. Fatty infiltration results in a hyperechoic appearance on ultrasound and diffuse hypoattenuation on computed tomography (CT) [10].

Radionuclide imaging may be employed to detect amyloid deposits in other tissues and organs, enabling assessment of disease extent and monitoring of treatment response [6,11].

If left untreated, amyloidosis may lead to progressive organ failure or even multi-organ failure [6]. Estimated survival in patients with AA amyloidosis depends on factors such as age, underlying disease aetiology, and treatment response, and ranges from approximately 79 months [12] to 133 months [13].

## 2. Case Presentation

A 28-year-old male patient noticed an increase in neck circumference in February 2024. His acquaintances also observed a change in the tone of his voice. His medical history included arterial hypertension, hyperuricaemia, ventriculoperitoneal shunt implantation shortly after birth, surgical repair of a myelomeningocele, bilateral congenital hip dislocation treated surgically in infancy, urostomy formation due to renal failure, and bilateral lower limb amputations at the level of the knees performed during childhood. The exact cause of amputations could not be established, as the patient does not possess medical records from that period. He had no prior symptoms or diagnosis of thyroid dysfunction. Thyroid function tests at admission were within normal ranges indicating euthyroidism (TSH: 0.965 µIU/mL, FT3: 2.38 pg/mL, FT4: 1.57 ng/dL), with negative thyroid autoantibodies (anti-TG: 19.30 IU/mL, anti-TPO: <9.00 IU/mL, TRAb: 1.43 IU/L). Serum calcitonin was 5.62 pg/mL, within the reference range.

At that time, the patient underwent a thyroid ultrasound (medical documentation unavailable). Based on abnormal sonographic findings, the examining physician urgently referred him to his general practitioner for further diagnostic evaluation. However, the patient discontinued follow-up due to fear of a potential malignancy.

Following the ultrasound, the patient reported decreased appetite alternating with episodes of intense hunger, unquantified weight loss, and persistent fatigue. In August, he developed profuse night sweats and fever, which lasted for approximately a month and then spontaneously resolved. He denied dyspnoea or pain in the thyroid region but reported progressive dysphagia.

In September 2024, the patient was admitted via the emergency department due to rapid neck enlargement and a fast-growing thyroid mass, raising suspicion of anaplastic thyroid carcinoma or lymphoma. A thyroid ultrasound performed during hospitalisation revealed a markedly enlarged gland located in the typical anatomical position, with a heterogeneous but predominantly normoechoic texture and normal vascularity on Colour Doppler imaging. The inferior poles of the thyroid were located above the level of the sternal notch, while the superior poles extended up to the mandibular angles. The lobes were too large to be measured with a linear probe. No focal lesions were identified, and the regional lymph nodes were not enlarged.

**Figure 1:**
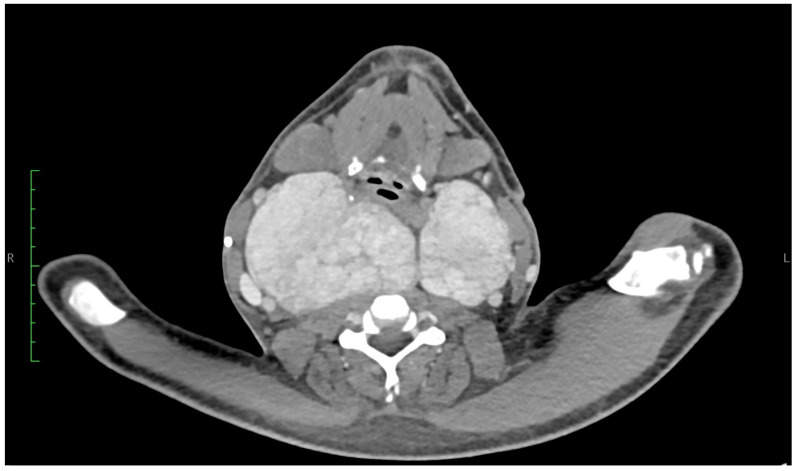
Neck CT. Frontal. The right thyroid lobe measures 74 × 89 × 140 mm (anteroposterior × right–left × craniocaudal). The upper pole reaches the level of the C1 vertebra, crosses the midline, and extends to the left side, displacing the larynx anteriorly and compressing the posterior right pharyngeal wall, resulting in narrowing of the airway. The left lobe measures 57 × 65 × 113 mm, with its upper pole reaching the level of the C2/C3 vertebrae. The lower poles of both lobes extend down to the superior border of the manubrium of the sternum. The isthmus measures up to 35 mm in width.

**Figure 2:**
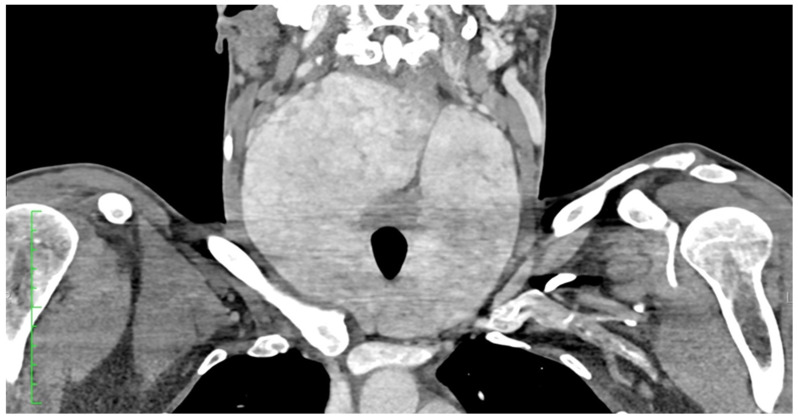
Neck CT. Axial. The contours of the thyroid gland are relatively smooth, causing displacement and compression of the surrounding structures: (1) bilateral displacement of the carotid vessels; (2) medial and rightward displacement of the oesophagus; (3) compression and slight rightward deviation of the trachea; (4) bilateral lateral displacement of the sternocleidomastoid muscles.

Laboratory investigations showed an abnormal protein electrophoresis profile, neutropenia, elevated beta-2 microglobulin, uric acid, and creatinine levels, as well as mild hyperkalaemia. Fine-needle aspiration cytology (FNAC) of the left thyroid lobe revealed a low-cellularity sample without atypical cells. Contrast-enhanced CT of the neck and chest showed a diffusely and significantly enlarged thyroid gland with strong but slightly heterogeneous enhancement [Figure 1 and Figure 2].

The right lobe measured 74 × 89 × 140 mm (anteroposterior × transverse × craniocaudal), with the upper pole reaching the level of vertebra C1. It crossed the midline to the left, displaced the larynx anteriorly, and compressed the posterior wall of the pharynx, causing luminal narrowing. The left lobe measured 57 × 65 × 113 mm, with the upper pole extending to the C2/C3 level. The inferior poles of both lobes reached the upper border of the manubrium. The isthmus measured up to 35 mm in width. The gland had relatively smooth contours and caused mass effect on adjacent structures: (1) bilateral displacement of the carotid vessels; (2) medial and rightward displacement of the oesophagus; (3) rightward deviation of the trachea; and (4) lateral displacement of the sternocleidomastoid muscles bilaterally. No enlarged cervical lymph nodes were noted.

The patient was discharged from the Endocrinology Department in good general condition with referral for outpatient endocrine follow-up. Due to ongoing rapid thyroid enlargement confirmed at the clinic, a repeat FNAC was performed one month later.

The repeat cytological specimen consisted mainly of cellular debris and CD45-negative cells without atypical antigen expression. Only morphotic elements of peripheral blood were observed, with no signs of atypia. The findings did not explain the rapid thyroid enlargement, and a surgical consultation was requested. The patient was referred for expedited admission to the Department of General and Oncological Surgery for total thyroidectomy.

**Figure 3:**
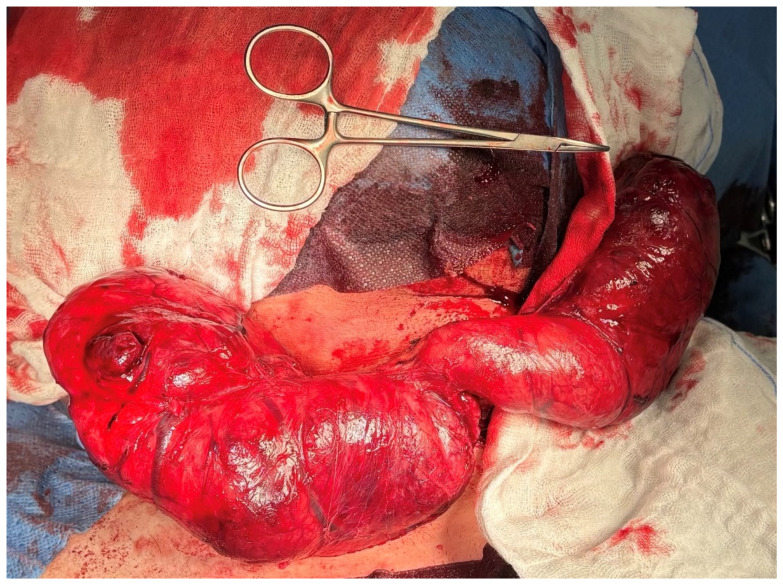
The thyroid gland resected during thyroidectomy measured 25 cm in the right lobe and 20 cm in the left lobe.

**Figure 4:**
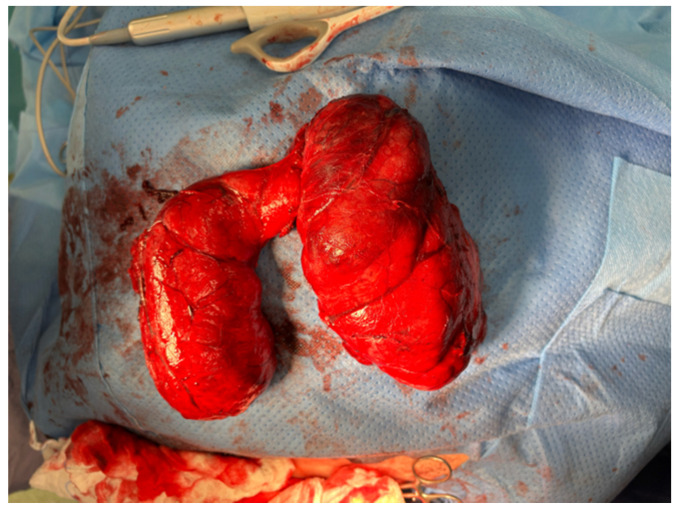
The thyroid gland resected during thyroidectomy.

On 5 November 2024, the patient was admitted and underwent total thyroidectomy via a Kocher incision, with preservation of the parathyroid glands and recurrent laryngeal nerves. Intraoperative neuromonitoring was utilised. A markedly enlarged thyroid gland was dissected, with the right lobe measuring 25 cm and the left lobe 20 cm. The superior poles and pyramidal lobe were ligated, and the parathyroid glands and recurrent laryngeal nerves were identified and preserved. The gland was removed en bloc, and haemostasis was achieved [Figure 3 and Figure 4].

**Figure 5:**
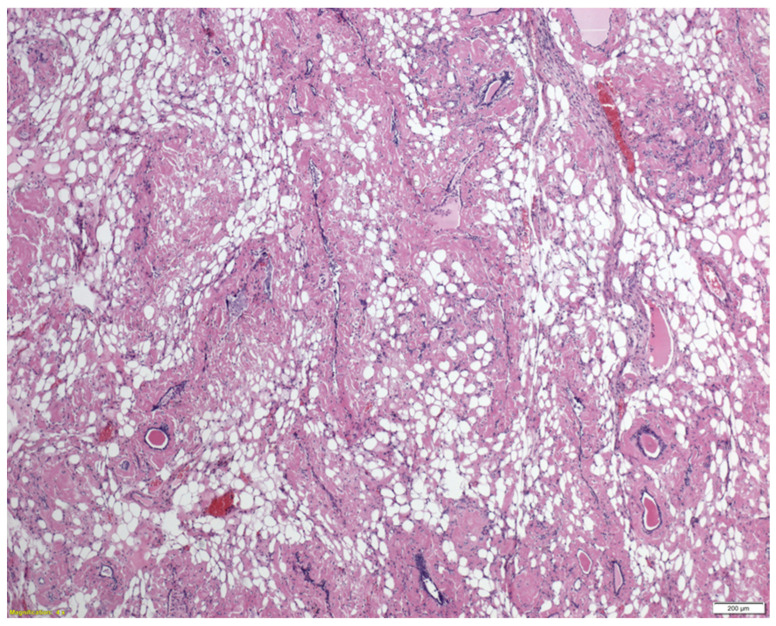
Thyroid follicles compressed and surrounded by the amyloid deposits. There is prominent adipocytic metaplasia of the stroma, which gives it a lacey appearance.

**Figure 6:**
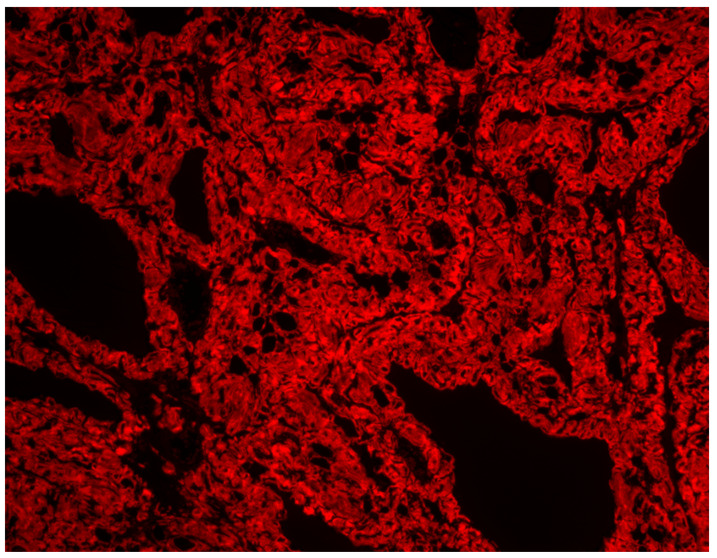
Congo red-stained thyroid tissue imaged with Texas Red-filtered fluorescence microscopy. The specificity of the staining has been confirmed by a control slide. Amorphous eosinophilic deposits exhibited a positive reaction with Congo red staining for amyloid.

Histopathological analysis revealed extensive deposition of amorphous material with accompanying atrophy of follicular cells. The amorphous eosinophilic masses were Congo red-positive, confirming the presence of amyloid. The findings were consistent with advanced thyroid amyloidosis, i.e., an amyloid goitre [Figure 5 and Figure 6].

During hospitalisation, transient elevation in creatinine levels was observed, which normalised following adjustment of medical therapy. The patient was discharged in good general condition on 11 November, after a 7-day hospital stay, with preserved phonation and no early postoperative complications. He subsequently presented to the endocrinology outpatient clinic with the histopathological report for further management. At the time of surgery, no clinical or radiological evidence of amyloidosis in other organs was present. Extended diagnostic work-up for systemic amyloidosis is currently in progress.

## 3. Discussion

Infiltrative thyroid diseases that should be considered in the differential diagnosis of a patient presenting with progressive neck enlargement include systemic sclerosis, haemochromatosis, sarcoidosis, chondrocalcinosis, amyloidosis [14], and malignant neoplasms. Historically, the Reimann classification was used to categorise amyloidosis based on its aetiology, dividing it into: (1) primary, (2) secondary, (3) tumour-associated, and (4) myeloma-associated amyloidosis [15,16].

**Figure 7:**
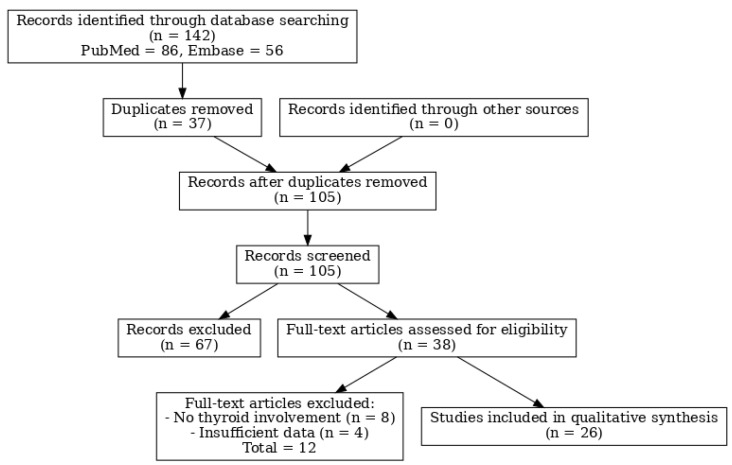
PRISMA 2020 flow diagram of the systematic literature review. The diagram illustrates the selection process of studies included in the qualitative synthesis. A total of 142 records were identified through PubMed and Embase searches. After removing duplicates (*n* = 37), 105 records were screened by title and abstract, resulting in the exclusion of 67 studies. Thirty-eight full-text articles were assessed for eligibility, of which 12 were excluded due to insufficient clinical details or lack of thyroid involvement. Finally, 26 studies were included in the qualitative synthesis.

Thyroid amyloidosis rarely leads to clinically palpable goitre [8,10,14], making it highly likely that many cases of mild thyroid enlargement due to amyloid deposits remain undiagnosed [8]. To better contextualise our findings, we conducted a systematic literature review in accordance with PRISMA guidelines. The search of PubMed and Embase (last updated 31 January 2025) identified 142 records, of which 26 studies met the inclusion criteria and were summarised in Table 1 (with the selection process shown in Figure 7).

Law et al. described five confirmed cases of amyloid goitre in patients aged 33, 44, 47, 52, and 85 years. All patients reported persistent hoarseness lasting for several years prior to the diagnosis of amyloidosis. The mean age at onset of initial compressive symptoms was 52.6 years [8], significantly older than our 28-year-old patient. One of the described patients reported fatigue; three had documented hypothyroidism, while one was diagnosed with thyrotoxicosis. By the end of follow-up, all patients exhibited thyroid dysfunction, either hypothyroidism or hyperthyroidism. One patient underwent near-total thyroidectomy with cricopharyngeal myotomy, which was complicated by bilateral vocal cord paralysis. In another case, a female patient was diagnosed with goitre at approximately 30 years of age. After 15 years, she underwent partial thyroidectomy to alleviate compressive symptoms; however, five years later, the goitre recurred and she developed dysphagia, leading to right lobectomy. Twelve years after the second procedure, she again presented with visible goitre enlargement and intermittent dysphagia [8]. These findings highlight the importance of regular post-thyroidectomy monitoring through imaging and laboratory assessment in the effective management and prevention of AA amyloidosis recurrence.

Patients with amyloidosis involving the head and neck region may present with a spectrum of clinical manifestations, including dysphonia, alterations in vocal timbre, and airway obstruction [17]. Rapid development of compressive symptoms such as dysphagia and orthopnoea indicates aggressive gland enlargement. This case underscores the importance of considering amyloidosis in the differential diagnosis of rapidly enlarging thyroid masses—even in young patients without typical comorbidities associated with the condition. In the case report “A rare case of primary thyroid amyloidosis” the patient presented with systemic manifestations of amyloidosis, renal failure, and multiple myeloma [18], further highlighting the uniqueness of our case. In both cases, histopathological diagnosis was confirmed with Congo red staining. Adipose tissue infiltration of the thyroid was also reported. Total thyroidectomy proved effective in alleviating compressive symptoms in both instances.

The presentation of amyloid goitre in a 28-year-old patient is highly unusual given the rarity of this condition in young individuals. Organ-limited amyloidosis is typically diagnosed in older adults, particularly in association with chronic diseases, multiple myeloma, or secondary amyloidosis linked to longstanding inflammatory conditions [19]. Due to the multiple comorbidities present in our patient, we conducted a thorough review of the available scientific literature and found no evidence of an association between the patient’s underlying conditions and the development of AA amyloidosis. Nagai et al. reported a case of a 25-year-old woman with amyloid goitre associated with vasculitis and transient thyrotoxicosis caused by the goitre. Physical examination revealed a diffuse, firm, elastic goitre with tenderness of the upper pole of the right thyroid lobe. Histopathological evaluation confirmed amyloid deposits and excluded subacute thyroiditis [20].

Ultrasonography allows visualisation of abnormal organ morphology, including both thyroid lobes, which are readily accessible for sonographic evaluation [21]. Pérez Fontán et al. described a 10-year-old boy with severe polyarticular juvenile idiopathic arthritis who developed a firm neck mass. Ultrasound revealed multiple hypoechoic lesions in the right thyroid lobe. CT imaging demonstrated several low-density thyroid nodules, and MRI showed markedly hyperintense thyroid masses on both T1- and T2-weighted sequences. Surgical biopsy revealed extensive amyloid deposition without haemorrhage or fatty infiltration [22].

In the case of our patient, attention must be paid to the potential impact of idiopathic amyloid deposition on prognosis and long-term involvement of other organs. Postoperative monitoring and extended diagnostic evaluation are essential following the early onset of idiopathic organ-limited amyloidosis. Further data collection is crucial to improve future outcomes and quality of life in patients presenting with this rare disease.

**Table 1:** Reported cases of thyroid amyloidosis/amyloid goitre. References: [8,14,18,20,22,23,24,25].

Author, Year	Age/Sex	Amyloid Type	Clinical Features	Treatment	Outcome
Law et al., 2013 [8]	33–85/5 pts	Secondary (AA)	Hoarseness, compressive symptoms, thyroid dysfunction	Thyroidectomy in most cases	Variable, vocal cord paralysis in one
Cannizzaro et al., 2018 [18]	58/F	Primary (AL)	Goitre, systemic amyloidosis, multiple myeloma	Total thyroidectomy	Symptom relief
Cohan et al., 2000 [14]	52/M	Secondary (AA)	Systemic amyloidosis secondary to ankylosing spondylitis	Total thyroidectomy	Stable
Pérez Fontán et al., 1992 [22]	10/M	Secondary (AA)	Neck mass, JIA	Surgical biopsy	NR
Nagai et al., 1998 [20]	25/F	Secondary (AA)	Goitre, vasculitis, thyrotoxicosis	Thyroidectomy	NR
Lari et al., 2020 [23]	54/F	Secondary (AA)	Goitre, compressive symptoms	Total thyroidectomy	Stable
Jakubović-Čičkušić et al., 2020 [24]	65/M	Secondary (AA)	Thyroid mass, systemic amyloidosis	Total thyroidectomy	NR
Gonzalez-Gil et al., 2024 [25]	72/F	Secondary (AA)	Amyloid goitre, systemic AA	Thyroidectomy	NR

## 4. Conclusions

AA amyloidosis occurs as a complication of chronic inflammatory conditions and may lead to serious complications, developing either insidiously—as is typical—or acutely. The kidneys are most commonly affected, often progressing to renal impairment. Other organs, including the heart, liver, and, as demonstrated in this case report, the thyroid gland, may also be involved, resulting in significant dysfunction.

This case highlights the importance of accurate and timely diagnosis of thyroid amyloidosis, particularly in light of its potential for rapid symptom progression. Early intervention and long-term follow-up are essential to ensure optimal treatment outcomes and ongoing patient management.

## Data Availability

No new data were generated in this study.

## Abbreviations

The following abbreviations are used in this manuscript:

CT	computed tomography
FNAC	Fine-needle aspiration cytology
AA	Amyloid A (Secondary Amyloidosis)
AP	Anteroposterior
CC	Craniocaudal
FNAB	Fine-Needle Aspiration Biopsy
MR	Magnetic Resonance
RL	Right–Left (transverse) dimension
USG/US	Ultrasonography
NR	Not reported
ESRD	End-stage renal disease
CRF	Chronic renal failure
RA	Rheumatoid arthritis
TB	Tuberculosis
AL	Amyloid light-chain
AH	Amyloid heavy-chain
Rx	Treatment
